# Anatomical Variant of Upper Humerus Notch in a Pediatric Case

**DOI:** 10.7759/cureus.38866

**Published:** 2023-05-11

**Authors:** Wahab A Gbadamosi, Lena Naffaa

**Affiliations:** 1 Diagnostic Radiology, Medical Center of Trinity, Trinity, USA; 2 Radiology Department, Nemours Children's Hospital, Orlando, USA

**Keywords:** normal variant, aggressive lesion, lytic bone lesion, humerus pseudotumor, upper humerus notch

## Abstract

When a medical image is requested for a particular indication, and a bony lesion is seen in a child, it causes anxiety to caregivers, unnecessary imaging costs, and unneeded biopsy. We present a complicated course of a five-month-old child who initially presented to the emergency room for prolonged cough and underwent a chest x-ray demonstrating clear lungs; however, a right humerus lytic lesion was identified. The child underwent multiple diagnostic imaging work-ups, which revealed a normal bone variation. This case report will describe a benign upper humeral notch variant with the goal to familiarize radiologists and clinicians with this entity and encourage them to obtain contralateral view radiographs to confirm bilaterality, prevent unnecessary advanced imaging as well added cost and anxiety to the parents.

## Introduction

The upper humeral notch is a bilateral symmetrical crescentic and irregular lytic lesion involving the proximal humerus cortex, which is related to the insertion of the shoulder joint capsule and belief to be a normal variant in a child [1]. However, this bony cortical irregularity can also be described in other etiology, such as glycogen storage disease, mucopolysaccharidosis, osteomyelitis, or neoplasm [1-2]. Therefore, recognizing normal variants from a pathological process on medical images prompts a diagnostic dilemma for clinicians when a benign disease can mimic an aggressive process in a symptomatic patient. Hence, this paper aims to describe a rare case of a normal upper humeral notch variant in a child because knowledge of this entity will prevent unnecessary parental anxiety and medical cost.

## Case presentation

**Figure 1:**
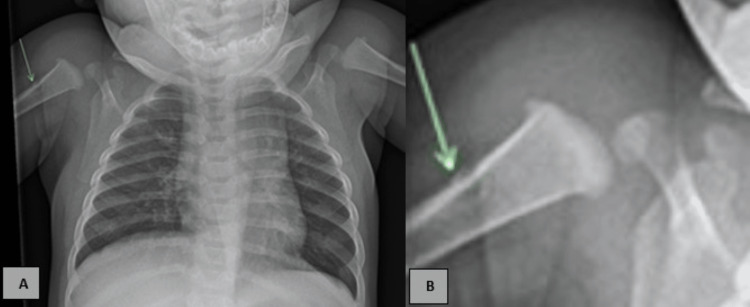
(A). Frontal chest radiograph demonstrates subtle cortical based lucency in the proximal right humeral metaphysis with irregular periosteal reaction (green arrow) with no other definite bony lesion seen. (B). Zoom down humerus image show ill-defined cortical irregularity.

**Figure 2:**
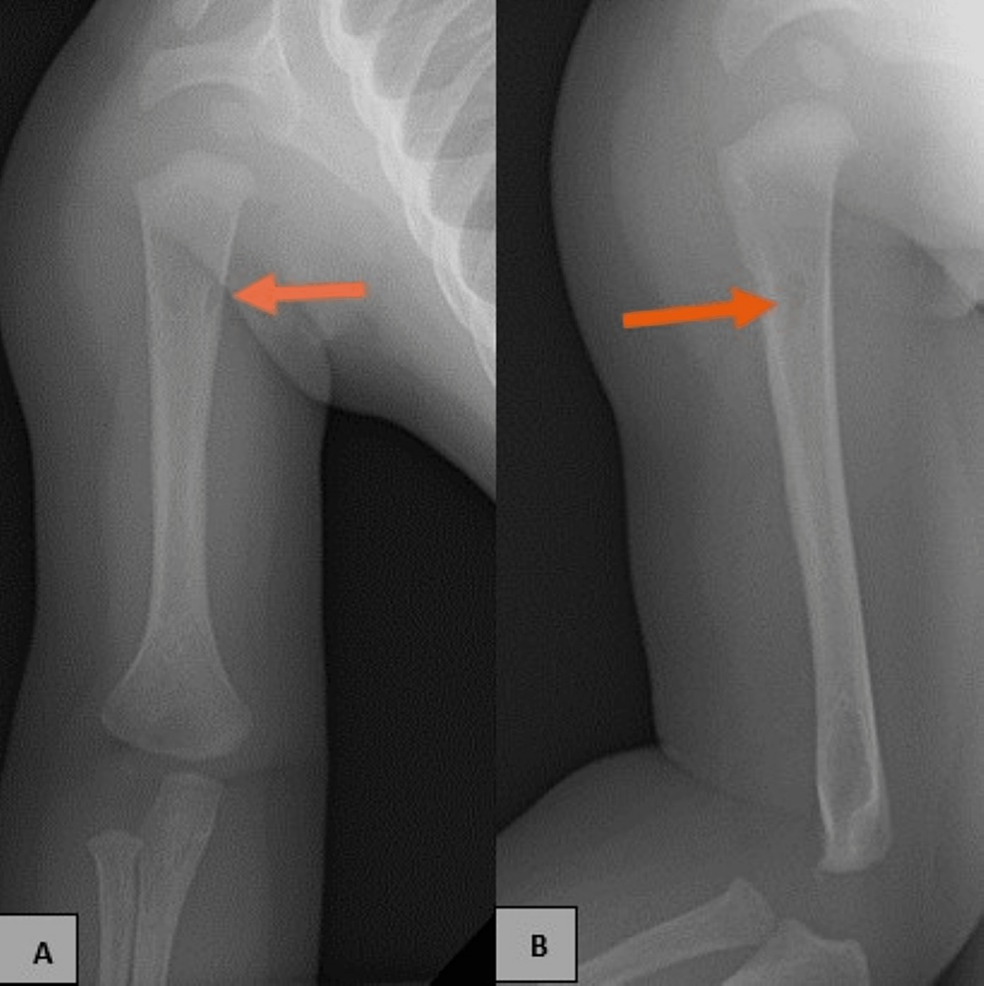
Frontal and external rotation view of right humerus (A and B) shows persistent subtle cortical-based lucency in the proximal right humeral metaphysis with irregular periosteal reaction (orange arrows).

We present a case of a five-month-old patient who presented to the emergency room for prolonged cough and fussiness with mildly elevated C-reactive protein done in an outside institution. Physical exam was not significant for fever (temperature of 36.4 °C or 97.5 °F); heart and respiratory rates were within normal limits. The patient appears alert but extremely fussy. Respiration was unlabored. His extremities were well-perfused with regular pulses. Musculoskeletal shows no tenderness, warmth, or erythema involving either upper extremity and has a full passive range of motion and symmetric appearance of both upper extremities. The remaining physical exam was non-contributory. Chest radiographs demonstrate no acute cardiopulmonary process: however, a subtle cortical-based lucency was seen in the proximal right humeral metaphysis with irregular periosteal reaction (Figure 1). A dedicated right upper extremity radiograph follow-up was obtained and confirmed the persistence of the lesion (Figure 2). The main differential diagnosis included a metastatic bone lesion, commonly from neuroblastoma at this age group versus osteomyelitis.

**Figure 3:**
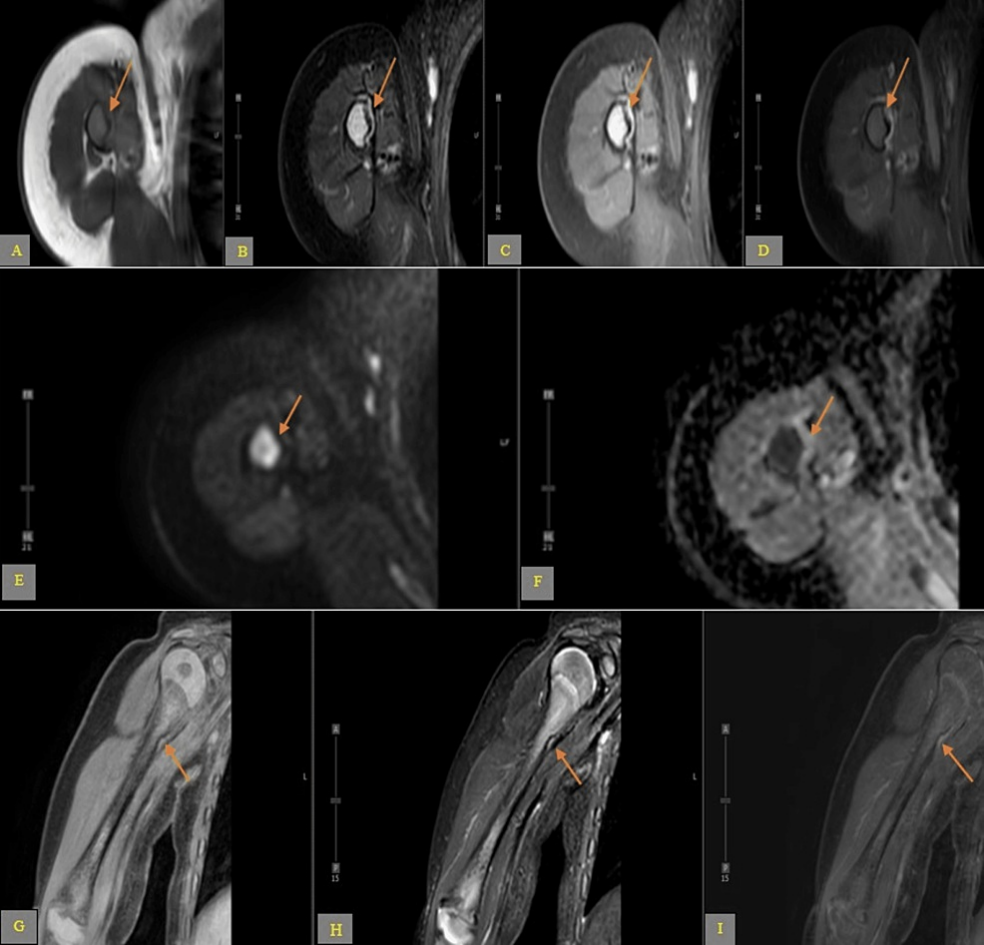
Series axial (A-F) and coronal (G-I) magnetic resonance imaging sequences. (A) T1 weighted imaging; (B) Fat saturated T2 weighted imaging; (C) Proton density; (D) T1-post contrast; (E) Diffusion weighted imaging; (F) Apparent diffusion coefficient; (G) Fat saturated T1 weighted imaging; (H) Short tau inversion recovery; (I) Post contrast subtraction sequence. These multiples magnetic resonance imaging sequences demonstrate a concave wavy appearing indentation of the medial meta-diaphysis cortex and periosteum of proximal right humerus with no discrete lesion, abnormal post-contrast enhancement, restriction diffusion, marrow, or soft tissue abnormality seen to correlate with the prior radiograph findings.

The differential diagnoses during the work-up included osteomyelitis, primary bone neoplasm, and neuroblastoma metastasis. Further work-up with ultrasound of the abdomen looking for an abdominal mass was negative. A decision was made to obtain magnetic resonance imaging (MRI) for additional morphology characteristics (Figure 3).

**Figure 4:**
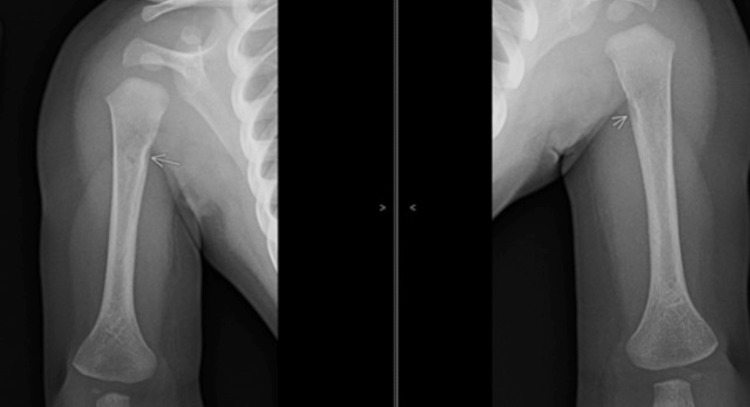
Follow-up frontal radiograph view of the bilateral humeri shows cortical notching involving the medial cortex of proximal bilateral humeral metadiaphysis suggesting a normal variation (arrows).

Given the benign imaging findings on magnetic resonance imaging, we recommended a follow-up radiograph of both arms for reassessment in four weeks. During the follow-up radiograph, the lesion on the right showed no significant change from the prior. In addition, the left upper arm radiograph showed a bony cortical wavy lesion involving the proximal humeral metadiaphysis, symmetrical to the lesion in the right proximal humerus (Figure 4), providing further support that this finding is most likely a normal anatomical variant.

## Discussion

The upper humeral notch is an asymptomatic, well-defined benign focal bony cortical irregularity defect affecting children’s proximal humeral meta-diaphysis [1]. It is a rare finding with an unknown incidence or prevalence within the pediatric population [1]. Due to this limitation, initial radiograph findings may generate clinical dilemmas, excessive medical imaging work-up, anesthesia, and biopsy when found incidentally. In our patient, abdominal ultrasound and magnetic resonance imaging were performed, which did not yield any malignant or infectious process; however, it did cause unnecessary anxiety to the parent, which could have been prevented.

The pathophysiology of the upper humeral notch is debatable, and multiple hypotheses regarding the origin of the humerus notch have been reported in the literature. Examples include infiltration of capsular attachments or sub-periosteal areas by normal cells or anatomic variations of benign character at tendon insertion [1,2]. However, most hypotheses of the cortical changes concluded to have emerged from chronic or subacute avulsion injuries [2,3].

The upper humeral notch can mimic other pediatric pathologies such as bony metastasis, osteoid osteoma, non-ossifying fibroma, glycogen storage disease, mucopolysaccharidosis, or osteomyelitis [2]. However, a pathological lesion rarely affects the bilateral proximal humerus symmetrically. Therefore, the bilaterality of this lesion irregularities in the proximal humerus and near the fibro-skeletal insertion site of the shoulder joint capsule can help supports the diagnosis of the upper humeral notch [1,2].

Radiographs and magnetic resonance imaging are the two key imaging modalities used in the pediatric population for bone lesions. In the upper humeral notch, imaging will demonstrate a geographically irregular crescentic notch with a margin appearing as either hazy, spiculated, sharp, or indistinct along the proximal humeral bony cortex [1]. It mostly involves the anteromedial insertion site of the latissimus dorsi and teres major muscle to the proximal humerus or can be anterolateral and corresponds to the pectoralis major insertion site [2-4]. When these findings involve the contralateral humerus tendon site attachment, it is most suggestive and indicative of this anatomical variant.

## Conclusions

The upper humeral notch is a focal cortical change that affects the proximal humerus and can mimic aggressive lesions. Hence, recognizing the upper humeral notch in pediatrics as an incidental benign finding is vital to prevent unnecessary imaging, biopsy, and related anesthesia while reducing parents’ anxiety and costs to the health care system.
